# Supplementary figures and images for: Timescales of motor memory formation in dual-adaptation
Source: PLoS Comput Biol. 2020 Oct 19;16(10):e1008373. doi: 10.1371/journal.pcbi.1008373 (PMC7595703; doi:10.1371/journal.pcbi.1008373)

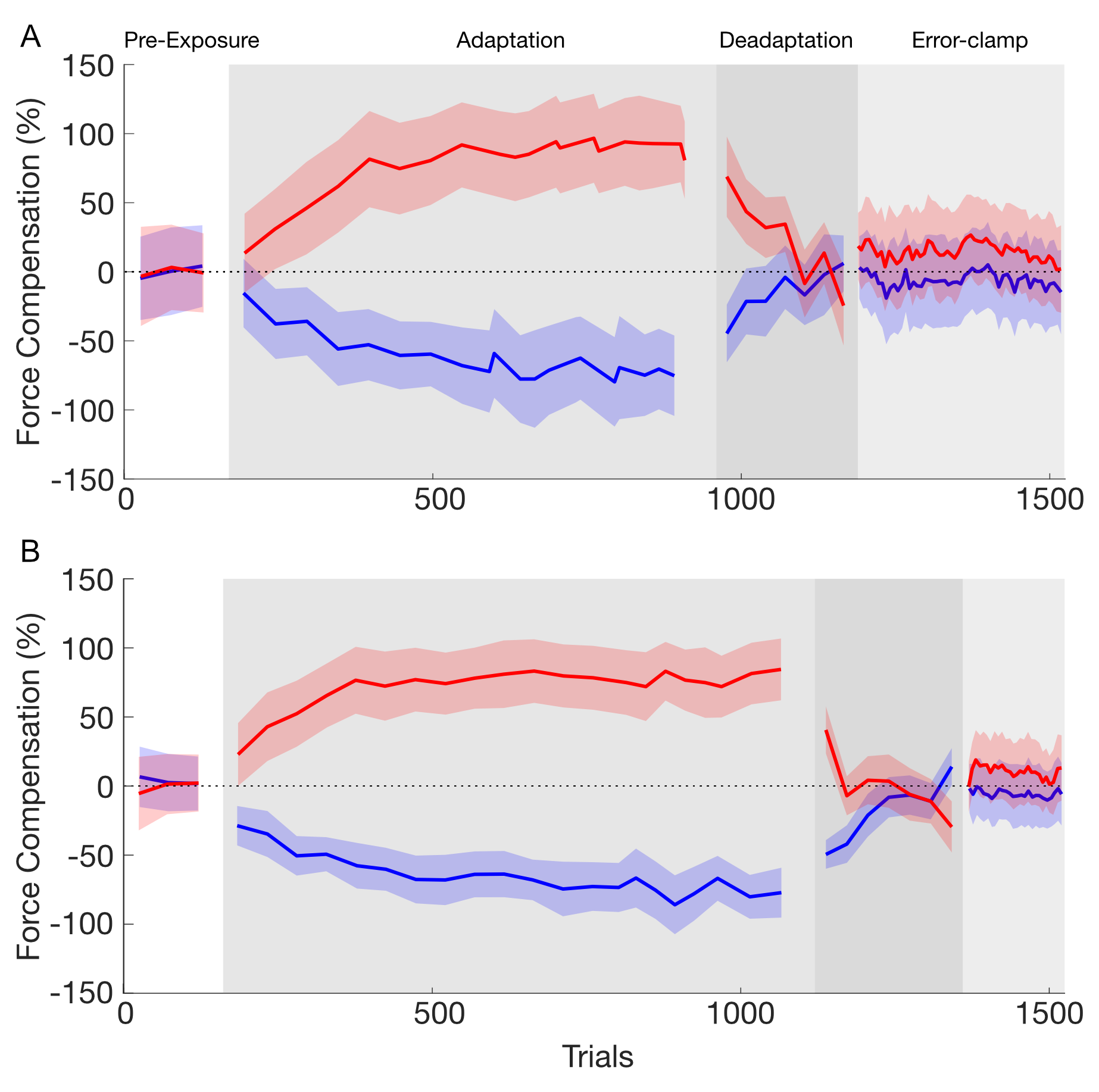

Supplement: S1 Fig — Mean of force compensation over pre-exposure (white), adaptation (grey), de-adaptation (dark grey) and error-clamp (light grey) phases. The mean force compensation across the two cues is not subtracted from the force compensation. The data of the contextual cue 1 (left visual workspace shift) and 2 (right visual workspace shift) are presented in red and blue lines, respectively. Shaded regions indicate the standard-error of the mean. A. Experiment 1. B. Experiment 2. (TIF) [file pcbi.1008373.s001.tif]

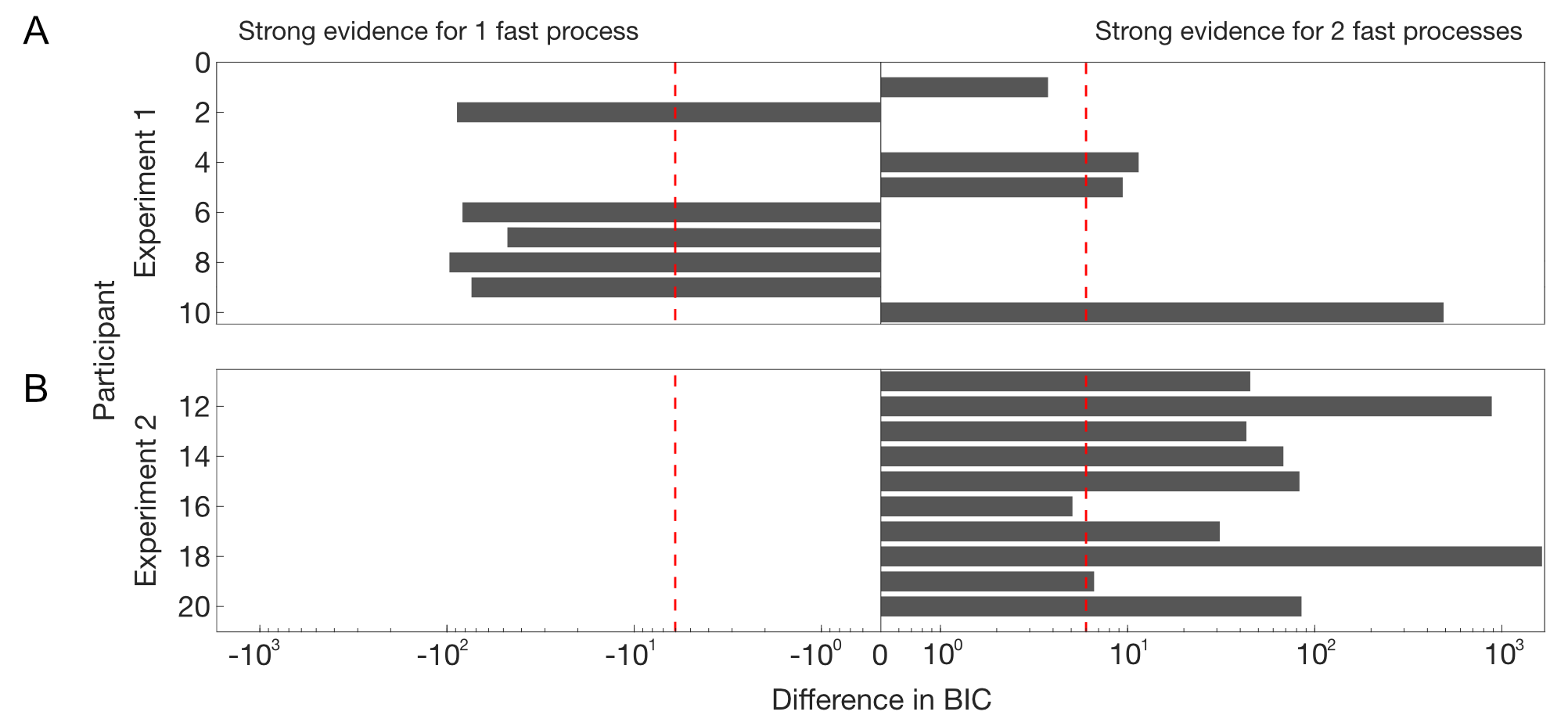

Supplement: S2 Fig — Bayesian Information criterion (BIC) differences between the one-fast-two-slow-dual-rate model (binary switch, left side) and the two-fast-two-slow-dual rate model (binary switch, right side). Improvements in BIC from 2 to 6, 6 to 10 and greater than 10 are considered as a positive, strong and very strong evidence of a model fitting better than the other models, respectively. The red dashed lines show a BIC difference of 6, indicating strong evidence towards one of the models. A. Experiment 1. B. Experiment 2. (TIF) [file pcbi.1008373.s002.tif]

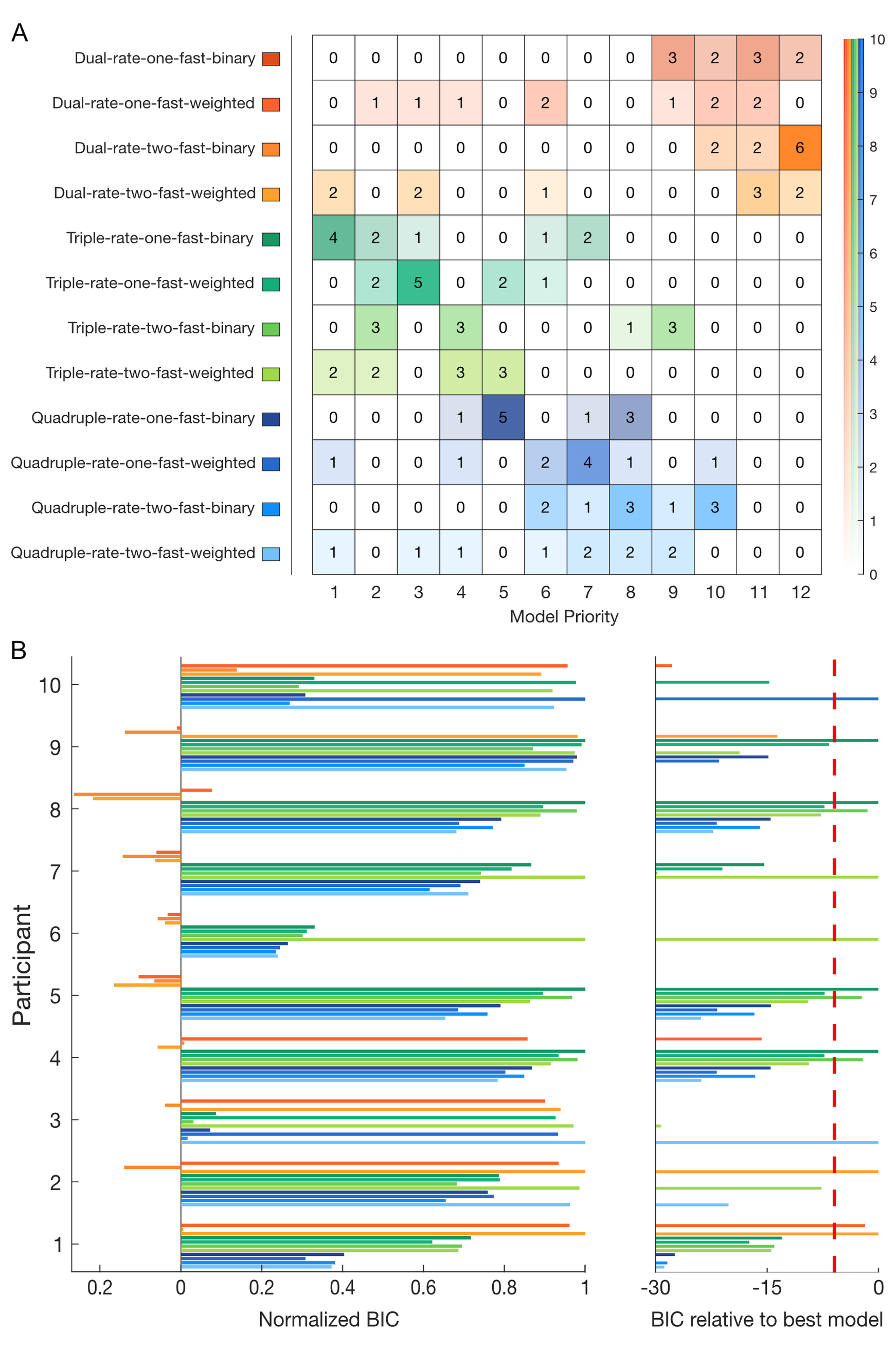

Supplement: S3 Fig — The fitting is run over the force compensation without subtraction of the mean between cues. A. Frequency table for each model (y-axis) by priority order (x-axis). This table represents the number of participants in which a given model was selected as the best-fit to the participant’s data by BIC from first chosen, to twelth (last) chosen. On the right-side, an opacity scale represents the number of participants. B. Individual BIC improvement for model comparison. The twelve models are differentiated by their improvement in BIC relative to the referent model (value of 0, left). For each participant, models are normalized to reveal the qualitative differences between each model and the best-fit model for that participant (value of 1). On the right side, the BIC improvement relative to the best-fit model is shown for the unnormalized values. Improvements in BIC from 2 to 6, 6 to 10 and greater than 10 are considered as a positive, strong and very strong evidence of a model fitting better than the other models, respectively. The red dashed line shows a BIC difference of 6 from the best-fitting model. (TIF) [file pcbi.1008373.s003.tif]

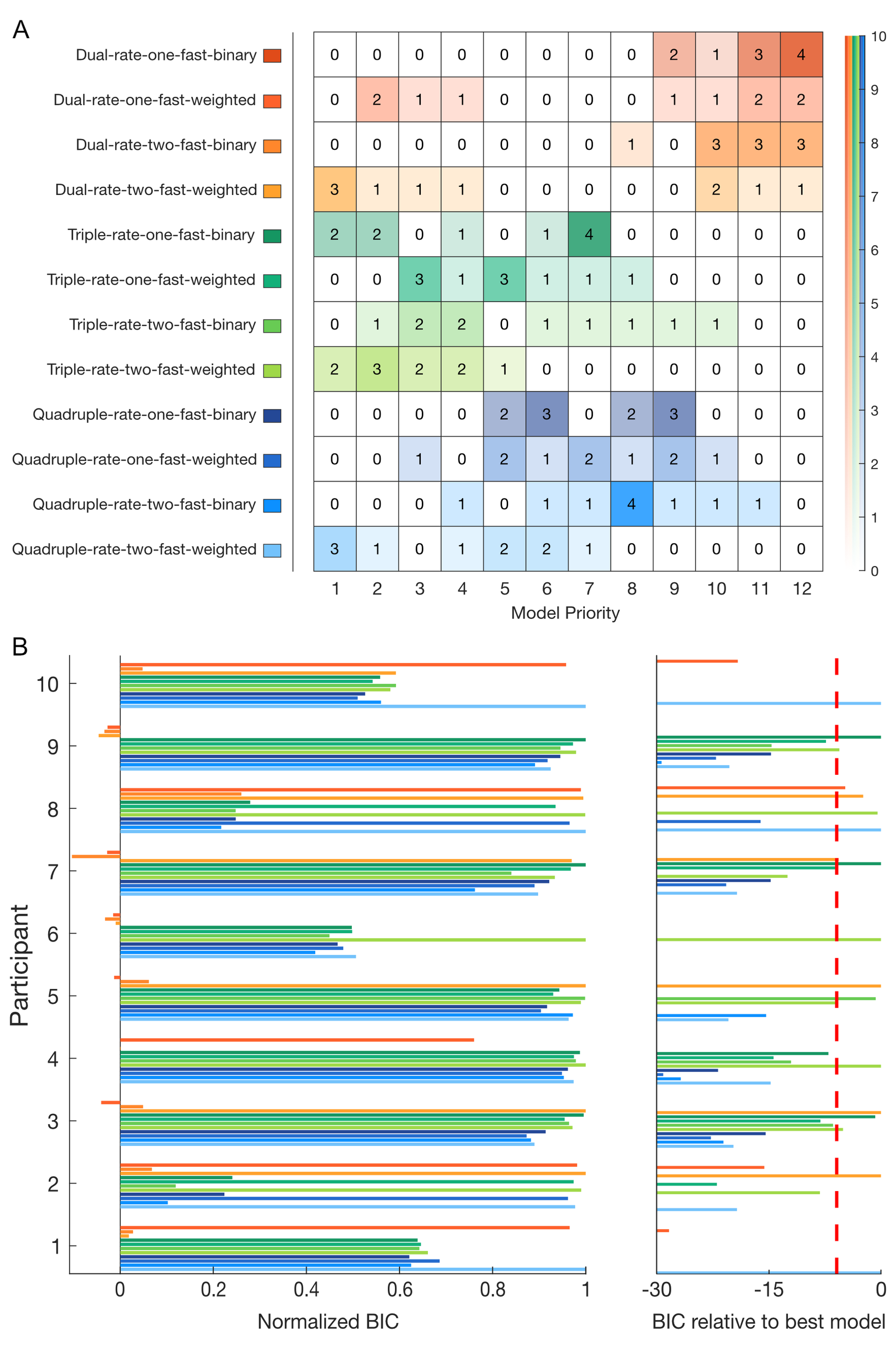

Supplement: S4 Fig — The fitting is run over the force compensation without subtraction of the mean between cues. Plotted as experiment 1. A. Frequency table for each model (y-axis) by priority order (x-axis). The right-side opacity scale represents the number of participants. B. Individual BIC improvement for model comparison. (TIF) [file pcbi.1008373.s004.tif]

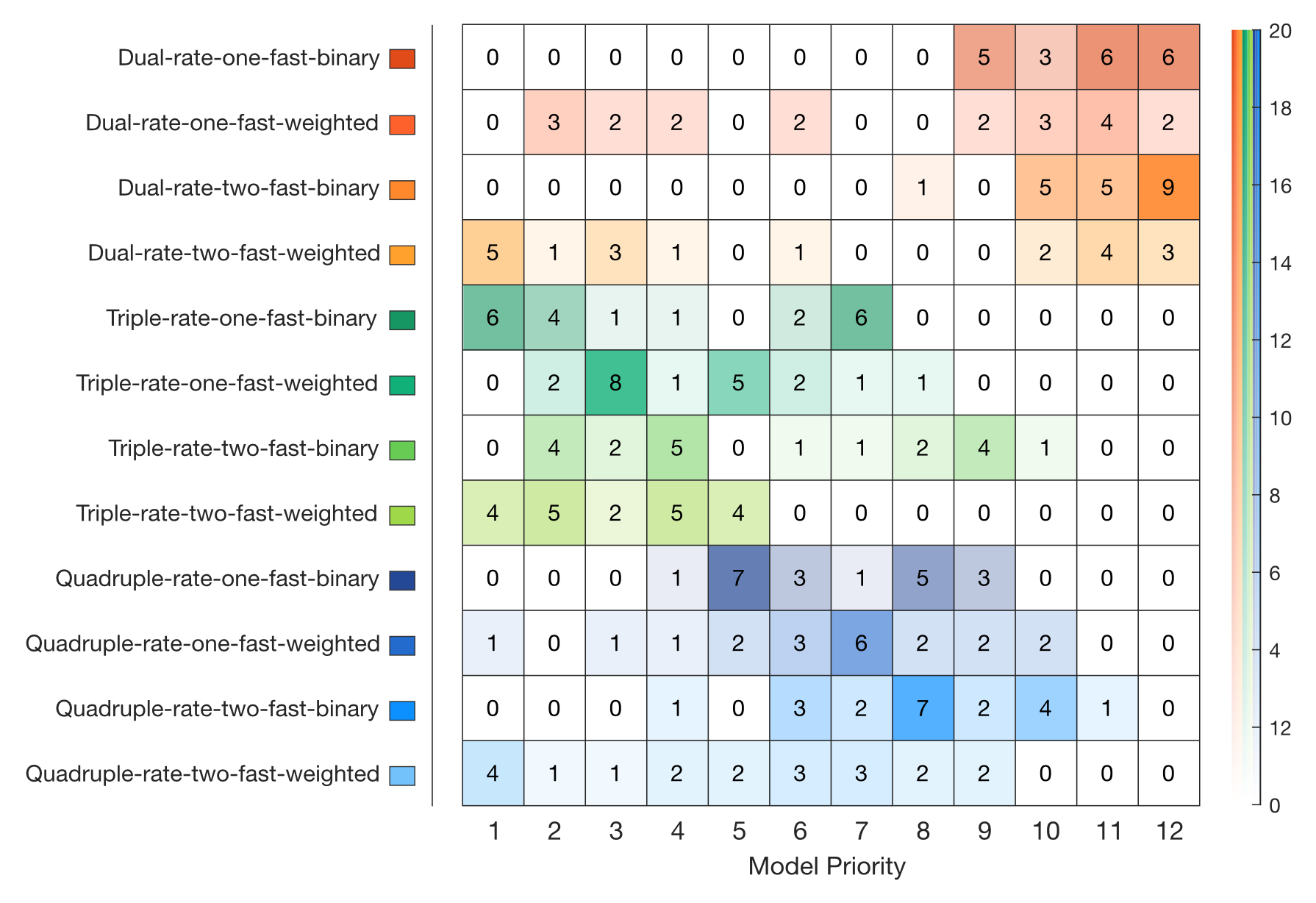

Supplement: S5 Fig — Frequency table of the individuals Bayesian Information criterion (BIC) model according to their priority (order of preference according to BIC improvement) across both experiments 1 and 2. The fitting is run over the force compensation without subtraction of the mean between cues. (TIF) [file pcbi.1008373.s005.tif]

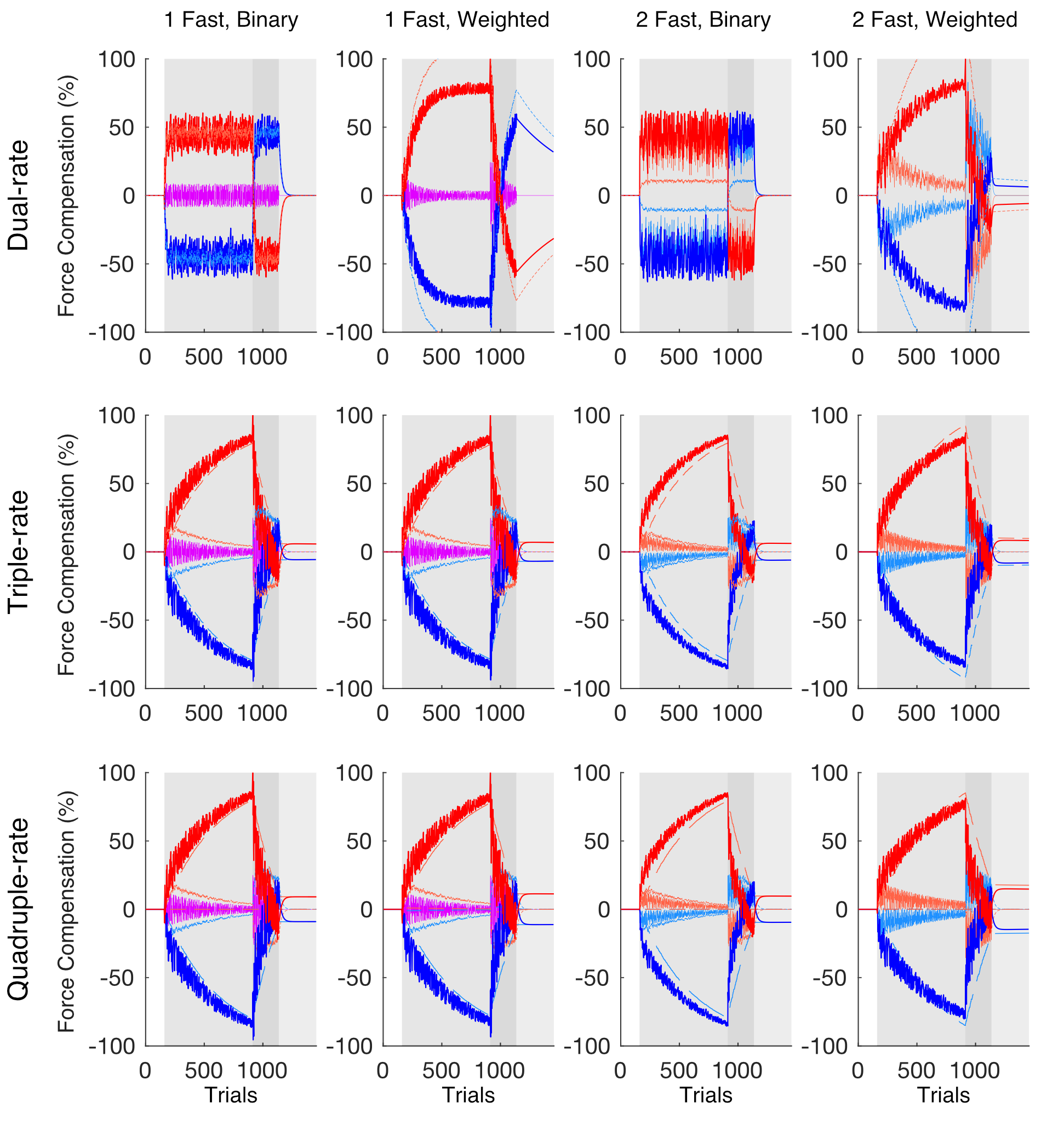

Supplement: S6 Fig — Here, the de-adaptation phase contained 220 trials (intermediate between experiment 1 and 2). The temporal phases are defined as pre-exposure (white), adaptation (grey), de-adaptation (dark grey) and error-clamp (light grey) phases. The data of the contextual cue 1 (left visual workspace shift) and 2 (right visual workspace shift) are presented in red and blue lines, respectively. The total output for each contextual cue (dark red and dark blue lines) is composed of the summation of each process (light red and light blue lines): hyperslow, ultraslow and slow processes (long, medium and short dashed lines respectively) and fast processes (solid lines). When a single fast process is shared between cues, this is represented with a magenta line. Note that spontaneous recovery is revealed for both contextual cues in the error-clamp phase for triple and quadruple-rate models. (TIF) [file pcbi.1008373.s006.tif]

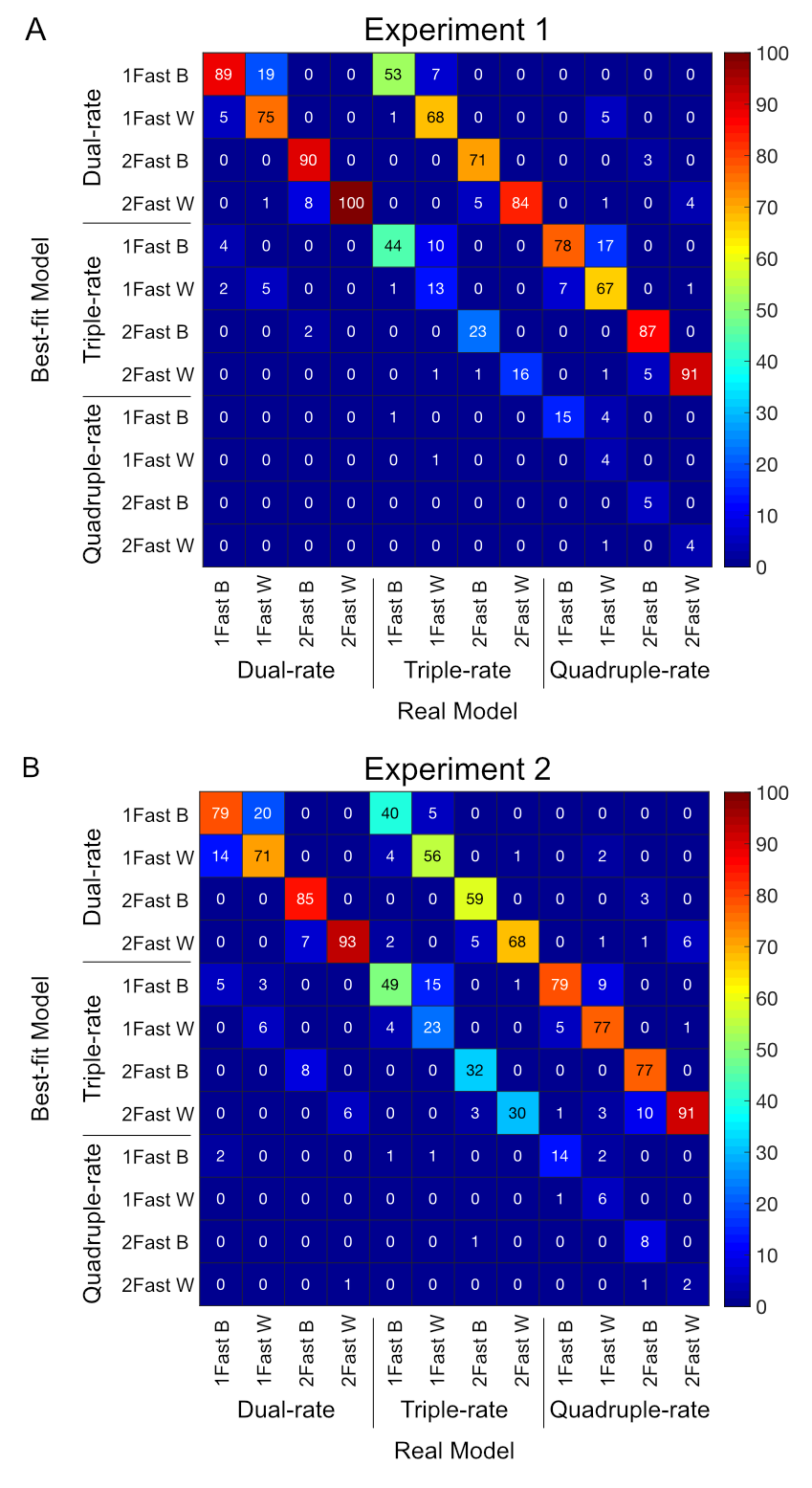

Supplement: S7 Fig — A. Experiment 1. B. Experiment 2. These matrices show the effect of prior parameter distributions on model recovery. Numbers denote the probability out of 100 repetitions (color scale) that data generated with model X are best fit by model Y, thus the confusion matrix represents p(bestfit model | real model). (TIF) [file pcbi.1008373.s007.tif]

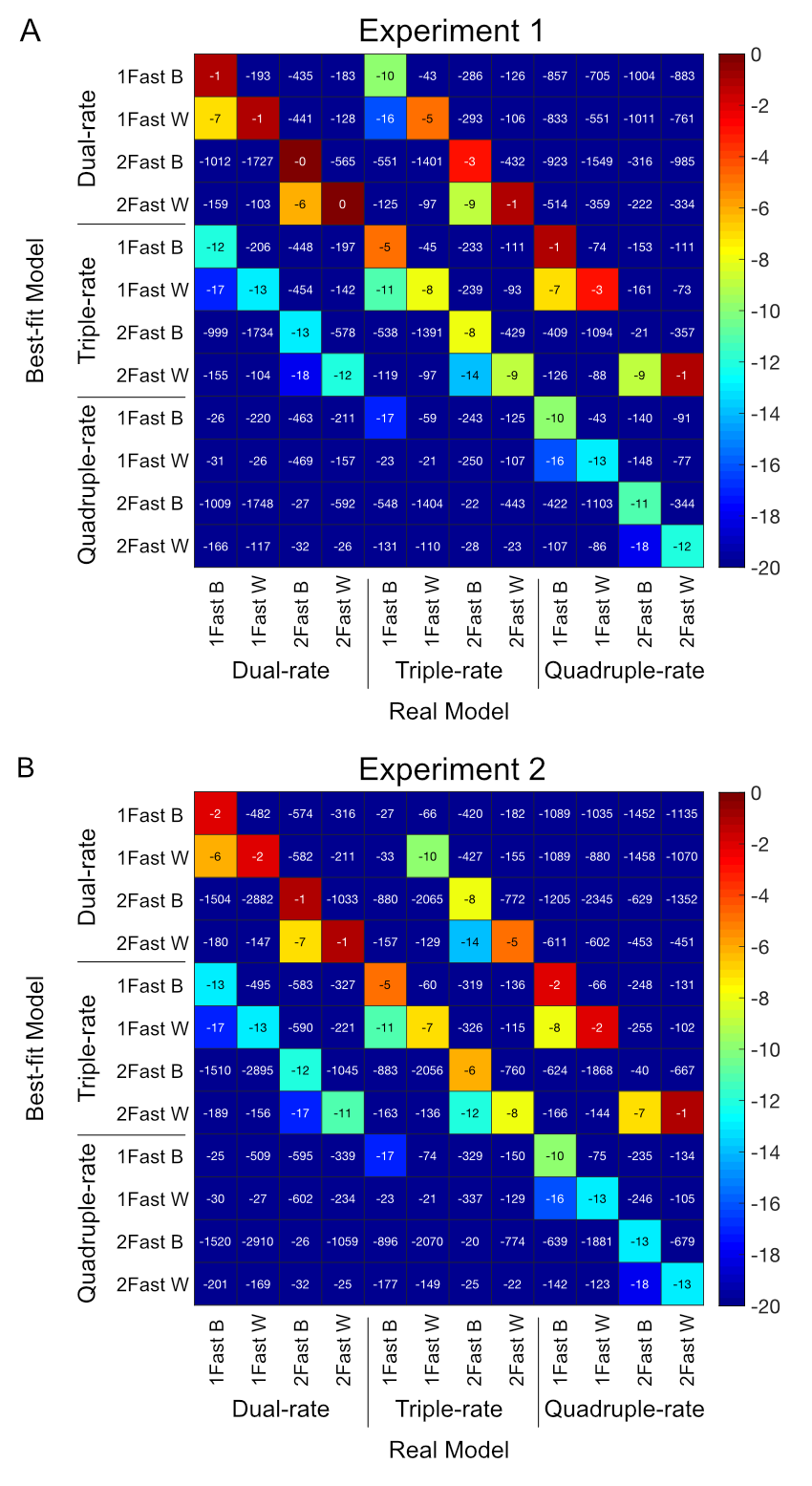

Supplement: S8 Fig — A. Experiment 1. B. Experiment 2. The values represent the average BIC differences relative to the best model. These matrices show the effect of prior parameter distributions on model recovery. Numbers denote the mean difference in BIC between this model and the best selected model across the 100 repetitions. If a specific model was selected as the best-fit model all 100 times, then this mean BIC difference would be zero. In all other cases, the values would be negative. This negative value corresponds to the average difference in BIC between each repetition and the best fit (value of 0). This table adds additional information on model ordering, as the confusion matrices (S7 Fig) do not take into account the magnitude of the BIC difference. We can see that the diagonal is always close to the best-fit model in terms of BIC even when it is not most often selected. (TIF) [file pcbi.1008373.s008.tif]
