# Supplementary material for: Timescales of motor memory formation in dual-adaptation
Source: PLoS Comput Biol. 2020 Oct 19;16(10):e1008373. doi: 10.1371/journal.pcbi.1008373 (PMC7595703; doi:10.1371/journal.pcbi.1008373)
Supplement: Attachment — Submitted filename: MForano_Answer_to_Reviewers.pdf [file pcbi.1008373.s010.pdf]

Timescales of motor memory formation in dual-adaptation - PLOS Comp Biol

## GENERAL COMMENTS

The reviewers were in general positive about the paper, but highlighted a number of concerns, mostly relating to the computational modeling and interpretation. In particular, the reviewers highlighted that the model fits are not presented in a transparent manner (only plotting BIC scores), despite it being seemingly straightforward to visualize predictions of this type of model. In addition, in order to improve the rigor of the modeling component of the paper, it would be worthwhile to include a model-recovery analysis using simulated data to determine how reliably the different models you consider can be discriminated based on the type of data collected in the experiments (see e.g. Wilson and Collins, eLife, 2019). In light of the reviews (below this email), we would like to invite the resubmission of a significantly-revised version that takes into account all the reviewers' comments.

We thank the editor for their positive comments and additional suggestions. We have now performed a model recovery analysis and added it in the manuscript.

We have taken into account the comments of all three reviewers and have re-performed the model fitting and parameter estimation, as well as added several additional figures in the Supporting Information. We thank all reviewers and the editor for their very helpful comments which have resulted in a much stronger manuscript.

# Reviewer #1:

Multi-rate models of adaptation have become an important tool used to understand the process of motor learning. Over the years, these models have undergone substantial adjustments, with the addition of multiple timescales, rules for adjusting error sensitivity over time, tweaks to allow for the formation of context-dependent memories, etc. Of these, one of the most influential on the field was the notion of a two-state model of learning: a model composed of a fast and slow timescale of adaptation. The reason why this model has been so successful is its ability to account for spontaneous recovery, or, the reemergence of an earlier memory in the absence of corresponding cues. About a decade ago, the Schweighofer lab expanded this dual-rate model, suggesting that adaptation is supported by a single fast state of learning, but multiple slow states. Here the authors challenge this idea, using a dual adaptation spontaneous recovery experiment. The authors show that behavior is only consistent with a model in which each context is supported by both a fast and slow state (i.e., the fast state is not shared across contexts). Therefore, the paper moves the field forward in demonstrating that fast timescales of learning, like slow timescales, are also context-dependent. Furthermore, the authors do a series of model comparisons to further evaluate the total number of slow and fast states that best describe adaptation, eventually settling on a model where in a given context, there is one fast state and two slow states of learning.
My overall feeling is that the authors do an excellent job of demonstrating that both fast and slow states of learning are context-dependent (which I find to be the most important conclusion of the paper), effectively invalidating the earlier model proposed by Schweighofer's group. However, as outlined below, I have some questions about the model comparison process, and if slight differences in their approach could yield a different conclusion regarding the total number of fast and slow states.

We thank the reviewer for their positive comments and suggestions. We have extensively revised the manuscript according to your comments.

**Major comments**

1. I understand that the authors subtracted off the mean compensatory response to the perturbation in order to recast their data so that there is equal adaptation within both contexts. I assume that this was necessary because there was unequal adaptation to the CW and CCW force fields. I see the benefit of doing so, as it allows the authors to fit subject behavior while minimizing the complexity of the model.

However, my concern is that any transformation of the original data may have an unintended effect on the model comparison process. In other words, is it possible that mean subtraction creates artificial features within the data that could bias the model comparison somehow away from some model classes, and towards the one fast, two slow state model?

We see the concern of the reviewer and have now included the same fitting and BIC results for both experiments without subtracting the offset in the Supporting Information. It can be

seen that the results provide a similar result but with much more variability. Importantly it can be seen that we still have strong preference for other models over the one-fast-two-slow state model previously proposed.

Along these lines, have the authors considered fitting the measured data without mean subtraction, but introducing an additional parameter that could account for the unequal extent of adaptation? For example, another parameter could be introduced that multiplies the error sensitivity terms for one context. Or perhaps this could be accounted for by allowing each context to possess two different "c" parameters.

As mentioned above, the Supporting information shows, the same models can be fit to the non mean adjusted force compensation data, without adding an additional parameter.

And while on this note, it would be good if the authors could show performance in both fields without mean subtraction, perhaps as a Supporting figure. And similarly, it would be helpful to show the best model fit to the data for each model class, or perhaps for the best dual-rate, triple-rate, and quadruple-rate model as a Supporting figure.

We have now included the performance in both fields and experiments without mean subtraction in the Supporting Information (Fig S2, S3 and S4). We have now added the best-fit simulation for every model type in the Supporting Information (Fig S5).

2. I appreciate that the authors provide BIC for each participant in Figs. 4 and 6. However, I am confused by the normalized BIC reports. For some participants, there does not appear to be a bar that reports the evidence for each model. For example, in Fig. 4B., for some participants, e.g., Participants 8 and 9, I do not see any lines that correspond to the dual rate model classes. Is this because the evidence for this model is so low that its normalized BIC improvement is near zero?

In the original manuscript, the x-axis limited its minimum value at 0, which means that only the positive individual BIC values were shown. This has been corrected in the current version and we now show the individual BIC of all the models including negative values.

One of the primary conclusions of the paper is that triple-rate models better describe behavior than dual-rate models. Certainly, this conclusion is well-supported by Figs. 4A and 6A. However, at the level of individual participants (1, 2, 3, 4, 6, 7, and 10 in Fig. 4B, and 1, 2, 3, 4, 5, 8, 9, and 10 in Fig. 6B) dual rate models appear to do as well as, if not better than the triple-rate models. To their credit, the authors do admit on L272, "that there is a variation in the best-fit models for each participant, with some better fit by the dual rate models and other

by the triple rate models". Given this, is there a concern that the superiority of the triple-rate model is being driven by a minority of participants? On this point, if it is the case that the dual rate models fit certain subjects quite poorly, but generally almost as good if not better than the triple-rate models, what is the reason for this? Is there some important behavioral difference between these two types of subjects?

We have redone the model fitting using stricter parameter constraints as suggested by the reviewers. Additionally, we now focus the model comparison only on the individual fits. Again, certain participants show a better fit for a lower order model than the triple rate model. However, now it can be seen that only 3 participants had a better fit with the dual rate model in experiment 1, and only a single participant in experiment 2. Most individual participants' results match well the overall findings of the experiments. Importantly we have now also included a model recovery analysis, which shows that our model fitting will often result in the lower order model producing a lower BIC, but that it consistently finds the correct number of fast processes and whether the cue should be binary or weighted. Based on these results, we think this provides even more evidence for higher order models. We now discuss this in more detail in the text.

3. The two fast triple rate model, and two fast dual rate model, are distinguished by the presence of an ultraslow state. In their section on "decay" (starting L402) the authors emphasize the possibility that this ultraslow state likely accounts for the persistence of the motor memory they observed in their terminal error-clamp period. However, given the model parameters reported in Table 1, it appears that the ultraslow state is best distinguished from the slow state not by its retention properties (which are no different than the slow state, as opposed to the ultraslow state reported in Inoue et al. 2015) but by its error sensitivity (which appears to be nearly an order of magnitude slower). To their credit, the authors admit that their attribution of error-clamp period decay to the ultraslow state is at odds with their best fit model parameters (L416). However, despite their honesty, it feels to me that attributing error-clamp period behavior to the ultra-slow process, is too speculative without additional analysis.

I do not follow the claim that because the decay rates in the decay block differ from those of the error-clamp period, that this is evidence for the existence of a slow versus an ultraslow state. It seems that a critical confound here is the differential contribution that the fast state might make to decay during each of these periods. Given the presence of this third state, why do the authors attribute differences in decay rates solely to the slow and ultraslow process?

We agree that this section may have been too speculative and have removed several sentences, and rewritten this section.

Along these lines, perhaps I have missed it, but it is unclear how the different retention curves

in Fig. 9 are simulated. The authors state on L405 that these are the simulations of a learning process with different retention rates ranging between 0.5 and 1.0. Which model does this learning process correspond to?

Importantly for this figure, we do not assume any specific learning process. Here we simply look at a range of decay rates and show what the decay would be compared to our data. In a real model, the decay would be a combination of these rates (e.g. a fast process, slow process and ultraslow process). Here, for exploring the relation of the data with different possible decay rates, the error-clamp phase was simulated. The decay was simulated such that $x^{n+1} = A \cdot x^{n}$ with the retention rate A being set as 0.5, 0.8, 0.9, 0.95, 0.98, 0.993, 0.997, 0.999 or 1. We chose a starting value corresponding to the level of learning of experiment 2 for comparison purposes. The idea here is to compare the experimental data with different decay rates in order to associate them with the rate of a slow, super slow of hyper slow rate. We have clarified this in the text.

**Minor comments**

1. I am a bit confused by the context parameter used for comparing the binary and weighted models. If I understand correctly, instead of using binary contexts 0 and 1, for their weighted models, they used contexts 0 and c, where c varies between 0 and 1. If this correct? Or did the authors model a system where the context parameter for one state is c and the other is 1-c as opposed to zero. It seems to me that the latter version is closer to their intent of having the model output a "weighted mixture between modules". Clarification here and within the text would be very helpful.
The weighted switch parameter was set as [c 1-c] where c varies between 0.5 and 1, with a summation of the two cue weights to 1. We have clarified this in the manuscript.

2. L327 – if I understand correctly, the authors highlight that at the end of deadaptation in Experiment 2, the additional deadaptation trials resulted in force profiles that had flipped their sign. In other words, forces for what was originally cue 1 had flipped to forces to what was originally cue 2. I am bit confused why the authors highlight this, because if anything this "flip" at the end of de-adaptation looks even stronger in Experiment 1 (Fig. 3E) than Experiment 2 (Fig. 5E). Am I misunderstanding something here?

The figures were incorrectly numbered in the original submission. This has been corrected in the current version.

3. Typo L788: A previous study [21] assumed the contextual switch… (delete "In" at the start of sentence).

Corrected.

4. The authors may want to rephrase their introduction a bit between L59-66. They start by

mentioning savings, interference, and spontaneous recovery. Then they introduce the two-state model giving the impression that this model accounts for all three phenomena. But at the end of the paragraph state that the two-state model only accounts for interference and spontaneous recovery. In addition to better clarifying this back-and-forth, the authors may want to consider citing:

a. Zarahn et al., 2008 for evidence that the two-state model does not account for savings.

b. Sing and Smith, 2010 when describing how the two-state model may account for interference

c. With reference to (b) the authors might also consider Lerner et al., 2019 (bioRxiv) for a more recent discussion of interference.

5. In terms of other citations, the authors might consider citing Heald et al., 2018 when describing dual adaptation and contextual cues.

We have revised the introduction and included the suggested citations.

## Reviewer #2:

This manuscript from Forano and Franklin tackles the question of timescales of motor memory formation during dual motor adaptation. Previous groups have shown that interference can be minimized and separate memories can be formed during simultaneous adaptation to opposing perturbations when participants are provided with appropriate contextual cues (Lee and Schweighofer 2009, Howard et al 2012, 2013, and 2015). This phenomenon was modeled by Lee and Schweighofer through expansion of a two-state multi-rate model of adaptation (Smith et al 2006), such that separate slow-learning processes were each assigned to one of the two perturbations, but only a single fast process adapted to opposing perturbations. Using simulation, two behavioral experiments, and model selection, the authors of the present manuscript propose a different computational architecture to that of Lee and Schweighofer. Specifically, they propose separate fast and slow processes for each perturbation, as well as a perturbation-specific "ultra-slow" process.

We thank the reviewer for the positive comments and suggestions. We have extensively revised the manuscript according to your comments.

Overall, I thought many parts of this manuscript were clearly written and the analyses were rigorous. However, I have major concerns regarding the narrow scope of the research question and thus the authors' attempt to frame their findings within the broader context of skill learning, as opposed to the more constrained realm of adaptation (re-calibration). At the end of the Introduction, the primary aim of the study was articulated as a question about whether or not the Lee and Schweighofer model could explain spontaneous recovery (the return to an adapted state during error clamp trials following washout of adaptation) during dual adaptation. While this question of interest, the link to skill learning (e.g., learning to ride a bicycle or ski) appears tenuous, especially in light of research showing that skill learning and recalibration are qualitatively different learning phenomena (Telgen et al 2014, Yang et al 2020 bioRxiv).

We also have published several papers looking at the learning of feedback control policies during movements, that both the Telgen and Yang papers discuss, but it is also important to consider that skill learning is not purely the result of learning a feedback policy. It involves many different aspects of the task including learning to produce specific patterns of external forces on the external world as in force field adaptation. I also think it is important to highlight the difference between visuomotor rotation (as in Telgen, 2014 and Yang, 2020 papers) and force field adaptation. Force field adaptation is not simply a recalibration in the same manner that visuomotor rotation is. Motor adaptation, particularly that of force field learning where the neural system learns the temporal pattern of muscle activation patterns to produce a specific pattern of endpoint forces, is clearly part of motor skill learning. Consider a simple task of a standing jump wherein one tries to maximize the height of the jump. Besides

simply increasing the strength of the muscles, one key factor is learning the temporal pattern of muscle activation that maximizes the jump height. We have made this point more clearly in the manuscript and outlined that this is only one part of overall motor skill learning. However, we appreciate the comment and have expanded on this point in the discussion, mentioning that this is only one of the many contributions to skill learning.

Other related Major Concerns follow:

- The addition of the error clamp trials to the AB experimental design was well-justified and had a clear link to the primary research question. However, I did not understand the justification for the subtle differences in experimental design between E1 and E2 and the main text did not provide a clear rationale. This hampered the overall impact of the manuscript as two similar sets of results and analyses were reported sequentially, but without any clear context for why both versions of the experiment were performed. For clarity, could the authors either provide a better justification for the two experimental designs and/or provide the rationale for combining the experimental results earlier so that readers only need to deal with one set of results? I believe this would help with clarity, as there were not independent pieces of information gained from both experiments.

We have now added text to explain why we conduct two studies. However one simple reason is simply that nowadays we should not rely on the output of a single experiment to make a claim, but should show that this result holds across multiple experiments. Here we have done this while varying key parameters of the design. Specifically, experiment 2 was added to examine the behavior in the error-clamp after a stronger de-adaptation phase. In experiment 2 we make sure that the participants are entirely deadapted from both cues and restrict the possibility of one cue to still be adapted toward the first learned task. Looking at the results, this second experiment is a stronger support for the previous experiment, knowing that even with stricter deadaptation, participants still show spontaneous recovery. We have clarified this in the text.

-I was surprised by the lack of explicit discussion of how the current results relate to the series of papers from Howard and Wolpert on forming separate internal models. I'm curious as to what the authors think regarding the generalizability of their findings and models to other methods of blocking interference, specifically dynamic contextual cues. This is in my mind of interest because the methods used in the current manuscript seem radically different than theirs, yet the phenomenon of interest is the same.

We have now included discussion of these papers in the current manuscript. We predict that these other types of cues would also produce similar temporal effects, but have not studied them in the current manuscript, so stating much more than we already do would be too

speculative at this point in time.

In addition, if we are to accept the findings of the Howard et al and Sheahan et al papers, don't their behavioral results already speak to the formation of two separate internal models? My point being that if we assume that each internal model has a slow and fast process, then the current proposition of two fast processes for two separate contexts is a natural corollary of what has already been shown in the literature. This is not to take away from the current findings and the more formalized approach, and of course, the introduction of a third ultra-slow process, but it does seem worth discussion in the text. This concern also points to the narrow scope of the manuscript.

We now make it clear in the manuscript early on that sufficient studies have already shown two separate internal models. However, importantly, previous findings have suggested the formation of two separate models driven by slow processes only. This is exactly what Lee and Schweighofer propose. Here we do not seek to justify the existence of independent motor memories, which has been already strongly stated many times. Instead we aim to explore the architecture of these independent memories and the structure of this adaptation by suggesting specific error-dependent fast processes instead of a general shared one. We have edited the text to make this clearer in the introduction.

-Since the bulk of the interpretation rests on the model-fitting procedures, could the authors provide figures showing model fits, at least for the models with characteristically different architectures, e.g., one-fast vs two-fast and two-slow vs two-fast and three-slow? I think such a figure would help contextualize the magnitude of the differences between the models, as I suspect the differences between two-fast two-slow vs two-fast three-slow would be fairly subtle as compared to one-fast vs two-fast. But without such a figure, the reader is only left to work with the BIC scores, which are informative, but not nearly as impactful as a figure with model fits could be.

We have now included the results for all of the best-fit models in the Supporting Information (Fig S5).

-While the figures showing the individual and group-averaged BIC results were both attractive and informative, I am concerned that too much emphasis is placed on group averaged scores, as there was no measure of across individual variance shown in Figs. 4 or 6. The mean improvements without the standard deviation or error tells only part of the story. Also, wouldn't it be justified to treat the BIC scores as any other dependent variable and subject them to inferential statistics, too? My point is that the BIC scores may benefit a great deal from contextualizing with figures of model fits and statistical tests. Since you are not testing different classes of models or radically different architectures, but rather different versions of multi-rate models that share a common algorithm, these suggestions would help

the reader form a more informed opinion regarding the robustness of the results.

Perhaps it was not clear, but part B of each of the figures showed the individual results for each subject. BIC results already provide statistical information about how different things are, and it would be inappropriate to perform an additional test on this data. Performing a statistical test on a Bayesian measure is inappropriate and does not appear helpful. In a way, it would be like performing a t-test on p-values. Instead the mean BIC values provide this information directly. Examining standard deviation or variance on the BIC values also does not make inherent sense as each subject will have a different fit depending on the variability of their data. To be statistically correct but to also address your e concern, we have now summarized the individual BIC into a frequency table. We have included the simulation results for all of the models as suggested.

On a related note, why does it appear that there are no normalized BIC scores for the orange and red models for participants 8 and 9 in Fig. 4B?

In the original manuscript, the x-axis limited its minimum value at 0, which means that only the positive individual BIC values were shown. This has been corrected in the current version and we now show the individual BIC of all the models including negative values.

-In terms of the writing, the Introduction and Results would benefit from more concrete details regarding some of the key concepts being introduced. For example, in the third paragraph, there is much discussion of contextual cues, but no delineation of specific cues that have been used in the literature and why some may be more effective than others (e.g., lead-ins or follow-throughs versus static color cues). Even as a member of the motor learning community, I was left wondering what you were specifically referring to. The overall effect is that the writing comes off as dry and likely impenetrable to someone outside of our small discipline. Also, as mentioned earlier, there does not appear to be a justification for E2, which also presents a barrier to the reader fully engaging with the study as currently presented.

We have revised the introduction to include more detailed descriptions.

-If you are proposing an ultra-slow process, shouldn't the parameter for A_us be constrained such that A_us > A_slow? I see in Table 1 that A_us is actually lower than A_slow. This doesn't seem to follow the logic of having separate processes acting on different timescales, i.e., learning slower and retaining more or vice versa. I'm not sure how much this would affect the results, but some explanation seems appropriate.

We agree with the reviewer. We have now introduced stronger constraints and have redone the analysis.

-Why was the mean force compensation across the two cues subtracted from the raw force

compensation? And why process only forces this way, as it appears kinematic errors were left alone?

We now include both the mean subtracted and non-mean subtracted (Supporting Information) for both the data and the model fitting. This was only done to the force compensation for better fit to the proposed model, as it is only fit to the force compensation data.

-Although the "stages" and "cues" used in the ANOVAs were clearly explained, there was no mention of what specific movement cycles were used in the analyses in the main text. This seems important to report as the effect sizes for spontaneous recovery do not appear very large.

The number of trials used for each stage: early exposure, late exposure, early de-adaptation and late de-adaptation is mentioned in the Material and methods *Statistics* lines 842-846: "For comparisons of kinematic error we compared across 4 stages (first 5 blocks in adaptation; final 10 blocks in adaptation; first 5 blocks in de-adaptation; final 5 blocks in de-adaptation). For comparisons of force compensation we compared across a different 4 stages (final 5 blocks in pre-exposure; final 10 blocks in adaptation; final 3 blocks of de-adaptation; all blocks of error-clamp phase)."

-The paragraph beginning on line 499 is a clear example of conflating adaptation with skill learning. Perhaps either flesh out the connection you would like to make in order to make the analogy to real world activities more convincing, or consider supplying appropriate caveats to your current interpretation.

We have addressed this issue in the comment above. Motor adaptation, particularly that of force field learning where the neural system learns the temporal pattern of muscle activation patterns to produce a specific pattern of endpoint forces, is clearly part of motor skill learning. Consider a simple task of a standing jump wherein one tries to maximize the height of the jump. Besides simply increasing the strength of the muscles, one key factor is learning the temporal pattern of muscle activation that maximizes the jump height. We have made this point more clearly in the manuscript and outlined that this is only one part of overall motor skill learning. We added this section to the discussion.

Minor concerns:

-The A and B panels are flipped in Figure 2.
Corrected.

-Caption for Figure 1 mentions contextual cues 1 and 2, but there has still been no mention of what these cues are.

Figure 1 shows two general simulations of the model dual-rate model from Lee & Schweighofer paper, assuming one or two fast processes. In this model the contextual cue switch parameter c, set as [0 1], does not take into consideration the type of cue. This model generally assumes that any contextual cue would have the same effect. We now mention our specific contextual cues where we first talk about the figure, although for modelling purposes this is arbitrary.

-There's a word or two missing in lines 545-548. This sentence overall is difficult to understand.

Corrected.

## Reviewer #3:

This study investigated the memory structure when adapting to the opposing dynamics during reaching movements. A previous study (Lee & Schweighofer, J Neurosci 2009) has proposed that the motor memory for the opposing visual rotation has a single fast and double slow motor memory. The authors tested the validity of this model by employing A-B-Error clamp experimental paradigm within dual-task adaptation. Contrary to the prediction by the previously proposed model, the authors observed the spontaneous recovery, clearly indicating the presence of two-fast memory states. They also fit various types of multi-rate models to the data and found the model with two fast, slow, and ultra-slow time constants best explained the data. This is an interesting study demonstrating a new aspect of motor memory structure, and the experiment and analysis were well designed. As described below, however, I would like to raise several issues, especially for the interpretation of the motor memory structure.

We thank the reviewer for their kind comments and suggestions. We have revised the manuscript according to these suggestions.

Major points

1) I was confused about the idea that motor memory has a discrete number of processes (e.g., two fast and slow processes). What does it mean? In the current experiment, the distance between the starting positions (i.e., visual workspace) was 20 cm. However, if the distance was 0 cm, different motor memories cannot be created. These facts indicate that different motor memories are likely to be created depending on the visual workspace. If it is the case, an infinite number of discrete processes seems necessary. The presence of two processes shown in this study is an epiphenomenon caused by the experimental design using just two contextual cues? How reasonable is assuming the memory has just two processes?

We completely agree with the reviewer and certainly do not claim that there are only two processes. Indeed our summary figure (Figure 10) shows multiple "motor memories." The goal of our study was to distinguish whether there was only a single fast process or multiple fast processes. The two processes here simply results from our two cues because we are using dual-adaptation as the reviewer points out. We have added a sentence in the results to make this clear from the beginning. Specifically we state:

"In dual adaptation, as there are only two force fields and two contextual cues, we will consider two separate learning processes, whereas the neural system must have a much larger capability."

2) The more natural idea would be to assume that the motor memories are composed of fast and slow (and ultra-slow) motor primitives (Thoroughman & Shadmehr, Nature 2000), and these motor primitives are recruited depending on visual workspaces. Then, the problem to be questioned here should be how the primitives are recruited rather than the number of

processes. I think that the model proposed in the present study is the approximation of such a motor primitive based model (i.e., the parameter ‘c’ is likely to reflect the way of encoding of visual workspace in the primitives). It seems a little bit odd to me that the motor memory has just two processes, and the contribution of each process is modulated with the visual cue. I do not think the authors need to change the whole story, but I would suggest that the authors mention the discussion on this issue.

We agree with these statements and are not claiming anything different. Our personal view is that these separate processes are made up of different motor primitives as in Thoroughman and Shadmehr (2000) or other related work. These are not mutually exclusive concepts, but much work in this area has investigated it at this level of abstraction, which is where we are describing it within this manuscript.

Minor points

1) Line 216: The comma should be removed?

Corrected.

2) Line 551: The “)” seems necessary after “[38]”.

Corrected.

3) Line 787: “In” should be removed?

Corrected.
