# Supplementary material for: Timescales of motor memory formation in dual-adaptation
Source: PLoS Comput Biol. 2020 Oct 19;16(10):e1008373. doi: 10.1371/journal.pcbi.1008373 (PMC7595703; doi:10.1371/journal.pcbi.1008373)
Supplement: Attachment — Submitted filename: MForano_Manuscript_Response_to_Reviewers.pdf [file pcbi.1008373.s011.pdf]

Timescales of motor memory formation in dual-adaptation - PLOS Comp Biol

REVISION

Reviewer 1

I thank the authors for their revisions to the manuscript, and for carefully considering the reviewers' comments. I do think aspects of the manuscript are improved (e.g., model priority analysis, model recovery analysis, and all data and analysis in the supplemental information). I remained convinced that the data and analysis clearly demonstrate the existence of two fast states, one per context. However, I still have some important concerns that I hope the authors might respond to, described below.

Thank-you very much for your helpful comments.

Major

1. In my original comments I noted my concern that for many participants, dual-rate models performed nearly as well or better than triple-rate models. In the revised manuscript, it is clearer that the triple-rate models are superior to dual-rate models, as well as quadruple-rate models at the level of individual participants (relative to the original manuscript). However, I am a bit concerned as to why the individual-level results appear to have changed so much from the original manuscript, to the revised manuscript. That is, in Experiment 1 of the original manuscript, it appeared that dual-rate models were better than triple rate models for participants 1, 3, 6, 7, and 10. And for Experiment 2, dual-rate models were superior for Participants 1, 2, 3, 4, 5, 8, and 10. In the revised manuscript, dual-rate models are best only for Participants 1, 5, and 10 for Experiment 1, and Participant 8 for Experiment 2 (as the authors note in their response to reviewers, and in their Results). So, for all data, dual-rate models were best for 12/20 participants in the original manuscript, but now only 4/20 participants.

It appears that these differences were caused by the addition of constraints on the parameter space for each model. I worry that tightening the parameter constraints may push the data towards the desired interpretation. My concern is exaggerated by inspection of Table 1. It appears that many parameters converged to the bounds used by the authors. For example, A_f often converged to 0.5, its lower bound. In addition, A_s often converged to 0.95, its lower bound. Moreover, B_f often converged to its lower bound of 0.1. And B_s often converged to 0.02, its lower bound.

Constraining the parameter space will make it so that each model's log-likelihood in the revised manuscript should be less than or equal to the log-likelihoods in the original manuscript. Therefore, what appears to have happened is that limiting the parameter space has eliminated "high-likelihood" dual-rate models, but has less affected the triple-rate models. Is my reasoning correct on this?

It is of concern that the dual-rate model fits should have changed so much. As far as I can see,

the only difference is that in the revised manuscript, B_s has a lower bound of 0.02, but could have approached 0 in the original manuscript. Is this the primary driver of differences between the revised manuscript and original manuscript? If so, should this (and other bounds) be altered?

We agree with the reviewer, as we were also concerned with adding additional constraints in our fitting, although this was what was suggested by reviewer 2. We therefore spent a great deal of time examining the effect of these constraints on the fits and much more importantly looking at what the dual-rate models were actually proposing as a solution. In the end, we believe that the constraints we currently have are needed for a fair comparison of the models, for a few reasons, the most important one which we outline here. We also agree that this is an important point, and have now added it to our discussion as a possible limitation of our study. We also think it is worth pointing out that we made quite an extensive revision of our analysis techniques in the last version, so not all of the changes in the results are due only to the addition of new constraints.

The major issue with the lack of constraints in the original submission became clear when we started investigating the parameters and plotting their independent contributions to the overall fitting in all of the twelve models. Specifically, dual rate models would end up fitting the data with a fast process and an ultra-slow process, in order to fit the data (avoiding the slow process completely). However, extensive previous work has measured the time-course of the slow process and estimated it to be far above 0.02. Using constraints which included all previously estimated values of the parameters is therefore a fair compromise, as the timescales of the models have a specific definition. That is, a slow process should have parameters within a specific range in order to be considered a slow process, otherwise it is not, by definition, a slow process. This is a very different approach from what is often done, but one we think is absolutely critical here. This is because the purpose of our study is not to simply ask how many timescales are necessary to fit the data. Instead, as we have extensive previous evidence that there is at least a fast and slow process, our purpose is to see whether these two timescales can explain our current data, or whether additional timescales are also required. As mentioned, we have now added these points to the discussion.

2. In light of Comment 1 above, without any experiment to specifically tease apart differences between dual-rate and triple-rate models, I still find it hard to put complete faith in these BIC comparisons. I think the authors may want to be more cautious in their conclusions about the superiority of the triple-rate model. At the very least, I feel the authors might include in their discussion that some participants did not show evidence of the ultra-slow process (and in fact, many do not if parameter constraints are relaxed). I commend them for doing so in their Results section on Lines 461-472. While the model recovery analysis is useful in interpreting why the dual-rate models may occasionally appear superior, I still think it may be helpful if the authors describe *why* some participants are fit much better by the triple-rate model than the dual-rate model or vice versa. If qualitative features of their behavior can explain why

dual-rate was good for some participants in the original manuscript, and triple-rate was good for others, this is an important piece of information to share with the reader. For example, is it possible that many participants do not have a lingering bias at the late stages of the final error clamp, and these are better described by a dual-rate model?

We have now investigated why specific participants are fit better by the dual-rate model and others by the triple-rate model, by focussing on these two groups of participants and looking for the differences in their adaptation. Specifically, we find differences in their initial rates of adaptation and de-adaptation, and outline how this changes the model fitting. It is also important to note that for the seven participants initially best-fit by the triple rate model, the triple-rate model fits much better than the dual-rate model, but for the three participants initially best-fit by the dual-rate model, there are no significant differences between the two models when fit across the participants simultaneously. We have added a paragraph and figure on this in the Results.

Minor

1. I like the model priority analysis that the authors added to the revised manuscript. However, I find the discussion of Figure 4 on Lines 283-297 very confusing. I would recommend that the authors consider re-framing their arguments here. Prior to this section, the manuscript focuses on comparison of the one-fast-two-slow model with the two-fast-two-slow model. It is hard to make this comparison at the level of individual participants using the model priority analysis in Fig. 4A, because it becomes lost in the sea of triple and quadruple rate models. The same goes for Fig. 6.

To help matters, I would recommend that the authors still report the group level results from Fig. 4A of the original manuscript (i.e. the mean BIC). There, it was clear that two-fast-two-slow is superior to one-fast-two-slow. I think the authors should continue to make this point (that two-fast-two-slow is better than one-fast-two-slow), before then opening up the analysis to consider model variants with a hyper-slow and ultra-slow state.

All in all, I would recommend keeping the mean BIC measure from Fig. 4A of the original manuscript, as well as the model priority analysis introduced in Fig. 4A of the revised manuscript (same for Fig. 6). The information provided in Fig. 4B of the revised manuscript is now somewhat redundant after the addition of the model priority analysis. In fact, it does not appear to be referenced in the text. The authors may want to consider moving it to SI. Of course, I leave this all to their discretion.

Then, I would recommend that the authors re-work the text on Lines 283-297. I think it will be easier to describe these results first from the group perspective (i.e., mean BIC) as in the original manuscript, and then use the model priority analysis as a way to confirm that the statistical comparison based on mean BIC was not somehow driven by a few participants.

We appreciate that this point got lost in the last version of the manuscript. We have now

revised the text, including much of the previous text but with a new figure to highlight this aspect. We did not include the previous mean BIC figure, as we realized that this figure is not a statistically appropriate method to show the data. However the new figure (Supporting Information Figure S2) demonstrates this point without the inclusion of other models.

2. I appreciate that the authors now show the data without mean subtraction in Fig. S1. However, I am surprised by the large differences in error bar size between Fig. S1 and Figs. 3&5. I suppose this could occur if the difference between CW and CCW adaptation is relatively consistent across participants, but the mean adaptation across both contexts is highly variable. However, I would ask that the authors check that they are displaying the error bars correctly in each figure.

The error bars are correctly displayed in each figure (the code used is the same). The differences in error bar size are caused by the participant's natural force shift to one side, reversed by counter-balancing half of the participants in the experiments. The natural force shift is produced as most participants move with a slight curvature to the leftwards direction for these movements with the right hand. This force shift is removed automatically when the mean cue is subtracted from the force compensation, and therefore the error bars are reduced in magnitude.

3. I think the manuscript is improved in its inclusion of the model recovery analysis. And the confusion matrix in Fig. S6 is great. However, it would also be helpful if the authors could summarize not only which models were selected in the recovery analysis, but by how much (in terms of the BIC difference).

We thank the reviewer for this suggestion, as we find the resulting figure quite useful. We have included a new figure in the Supporting information (Fig S8) which shows the average BIC difference of each model compared with the best-fit model.

Reviewer 2

I found the revised manuscript to be much clearer and easier to read than the original submission, and I appreciate the changes the authors made based on our suggestions. The additional discussion of previous literature related to contextual interference and ideas surrounding multiple paired models helped me better contextualize the current findings. In addition, the parameter recovery analysis and new figures strengthened the authors' overall interpretation of their findings.

Thank-you very much for your helpful comments.

Below are additional suggestions and minor comments for the authors to consider:

- I suggest redoing Fig. S5 and including a revised version in the main text. Due to the compression of the x-axes, the simulations are not nearly as clear or informative as they could be. Have the authors considered selecting a subset of their models and showing them in a more expanded form in the main text? The remaining models (or all of them) could remain a supplementary figure, with fewer columns and more rows.
The simulation of three models were added in the main manuscript in order to examine further the difference between timescales (Fig 10).

Minor:

- The font in the tables is too small and difficult to read without zooming in.
Corrected.

- The language used to describe best-fit models was not always clear. For example, lines 285-286: "…out of ten participant, nine participants equally chose the…" The italicized portion is what I'm referring to. Also, here and throughout, the authors describe participants "choosing" their models. Wouldn't it be more accurate, and less idiosyncratic, to write in these cases something like "…the X model provided the best fit to a out of b participants."
Corrected.

- L381: I would replace "deeper" with "greater" or "more". I wasn't clear about what "deeper" meant until looking at the figure.
Corrected.

- L551: This sentence was difficult to parse, especially the reference to "in the concept of single adaptation".
Corrected.

Reviewer 3
I am satisfied with the authors' explanation regarding the interpretation of multiple motor memories. I have no further concerns.

Thank-you very much for your helpful comments.
