# Supplementary material for: Timescales of motor memory formation in dual-adaptation
Source: PLoS Comput Biol. 2020 Oct 19;16(10):e1008373. doi: 10.1371/journal.pcbi.1008373 (PMC7595703; doi:10.1371/journal.pcbi.1008373)
Supplement: Attachment — Submitted filename: comments.docx [file pcbi.1008373.s009.docx]

Multi-rate models of adaptation have become an important tool used to understand the process of motor learning. Over the years, these models have undergone substantial adjustments, with the addition of multiple timescales, rules for adjusting error sensitivity over time, tweaks to allow for the formation of context-dependent memories, etc. Of these, one of the most influential on the field was the notion of a two-state model of learning: a model composed of a fast and slow timescale of adaptation. The reason why this model has been so successful is its ability to account for spontaneous recovery, or, the reemergence of an earlier memory in the absence of corresponding cues. About a decade ago, the Schweighofer lab expanded this dual-rate model, suggesting that adaptation is supported by a single fast state of learning, but multiple slow states. Here the authors challenge this idea, using a dual adaptation spontaneous recovery experiment. The authors show that behavior is only consistent with a model in which each context is supported by both a fast and slow state (i.e., the fast state is not shared across contexts). Therefore, the paper moves the field forward in demonstrating that fast timescales of learning, like slow timescales, are also context-dependent. Furthermore, the authors do a series of model comparisons to further evaluate the total number of slow and fast states that best describe adaptation, eventually settling on a model where in a given context, there is one fast state and two slow states of learning.

My overall feeling is that the authors do an excellent job of demonstrating that both fast and slow states of learning are context-dependent (which I find to be the most important conclusion of the paper), effectively invalidating the earlier model proposed by Schweighofer’s group. However, as outlined below, I have some questions about the model comparison process, and if slight differences in their approach could yield a different conclusion regarding the total number of fast and slow states.

**Major comments**

1. I understand that the authors subtracted off the mean compensatory response to the perturbation in order to recast their data so that there is equal adaptation within both contexts. I assume that this was necessary because there was unequal adaptation to the CW and CCW force fields. I see the benefit of doing so, as it allows the authors to fit subject behavior while minimizing the complexity of the model.

However, my concern is that any transformation of the original data may have an unintended effect on the model comparison process. In other words, is it possible that mean subtraction creates artificial features within the data that could bias the model comparison somehow away from some model classes, and towards the one fast, two slow state model?

Along these lines, have the authors considered fitting the measured data without mean subtraction, but introducing an additional parameter that could account for the unequal extent of adaptation? For example, another parameter could be introduced that multiplies the error sensitivity terms for one context. Or perhaps this could be accounted for by allowing each context to possess two different “c” parameters.

And while on this note, it would be good if the authors could show performance in both fields without mean subtraction, perhaps as a supplementary figure. And similarly, it would be helpful to show the best model fit to the data for each model class, or perhaps for the best dual-rate, triple-rate, and quadruple-rate model as a supplementary figure.

2. I appreciate that the authors provide BIC for each participant in Figs. 4 and 6. However, I am confused by the normalized BIC reports. For some participants, there does not appear to be a bar that reports the evidence for each model. For example, in Fig. 4B., for some participants, e.g., Participants 8 and 9, I do not see any lines that correspond to the dual rate model classes. Is this because the evidence for this model is so low that its normalized BIC improvement is near zero?

One of the primary conclusions of the paper is that triple-rate models better describe behavior than dual-rate models. Certainly, this conclusion is well-supported by Figs. 4A and 6A. However, at the level of individual participants (1, 2, 3, 4, 6, 7, and 10 in Fig. 4B, and 1, 2, 3, 4, 5, 8, 9, and 10 in Fig. 6B) dual rate models appear to do as well as, if not better than the triple-rate models. To their credit, the authors do admit on L272, “that there is a variation in the best-fit models for each participant, with some better fit by the dual rate models and other by the triple rate models”. Given this, is there a concern that the superiority of the triple-rate model is being driven by a minority of participants?

On this point, if it is the case that the dual rate models fit certain subjects quite poorly, but generally almost as good if not better than the triple-rate models, what is the reason for this? Is there some important behavioral difference between these two types of subjects?

3. The two fast triple rate model, and two fast dual rate model, are distinguished by the presence of an ultraslow state. In their section on “decay” (starting L402) the authors emphasize the possibility that this ultraslow state likely accounts for the persistence of the motor memory they observed in their terminal error-clamp period. However, given the model parameters reported in Table 1, it appears that the ultraslow state is best distinguished from the slow state not by its retention properties (which are no different than the slow state, as opposed to the ultraslow state reported in Inoue et al. 2015) but by its error sensitivity (which appears to be nearly an order of magnitude slower). To their credit, the authors admit that their attribution of error-clamp period decay to the ultraslow state is at odds with their best fit model parameters (L416). However, despite their honesty, it feels to me that attributing error-clamp period behavior to the ultra-slow process, is too speculative without additional analysis.

I do not follow the claim that because the decay rates in the decay block differ from those of the error-clamp period, that this is evidence for the existence of a slow versus an ultraslow state. It seems that a critical confound here is the differential contribution that the fast state might make to decay during each of these periods. Given the presence of this third state, why do the authors attribute differences in decay rates solely to the slow and ultraslow process?

Along these lines, perhaps I have missed it, but it is unclear how the different retention curves in Fig. 9 are simulated. The authors state on L405 that these are the simulations of a learning process with different retention rates ranging between 0.5 and 1.0. Which model does this learning process correspond to?

**Minor comments**

1. I am a bit confused by the context parameter used for comparing the binary and weighted models. If I understand correctly, instead of using binary contexts 0 and 1, for their weighted models, they used contexts 0 and c, where c varies between 0 and 1. If this correct? Or did the authors model a system where the context parameter for one state is c and the other is 1-c as opposed to zero. It seems to me that the latter version is closer to their intent of having the model output a “weighted mixture between modules”. Clarification here and within the text would be very helpful.

2. L327 – if I understand correctly, the authors highlight that at the end of deadaptation in Experiment 2, the additional deadaptation trials resulted in force profiles that had flipped their sign. In other words, forces for what was originally cue 1 had flipped to forces to what was originally cue 2. I am bit confused why the authors highlight this, because if anything this “flip” at the end of de-adaptation looks even stronger in Experiment 1 (Fig. 3E) than Experiment 2 (Fig. 5E). Am I misunderstanding something here?

3. Typo L788: A previous study [21] assumed the contextual switch… (delete “In” at the start of sentence)

4. The authors may want to rephrase their introduction a bit between L59-66. They start by mentioning savings, interference, and spontaneous recovery. Then they introduce the two-state model giving the impression that this model accounts for all three phenomena. But at the end of the paragraph state that the two-state model only accounts for interference and spontaneous recovery. In addition to better clarifying this back-and-forth, the authors may want to consider citing:

a. Zarahn et al., 2008 for evidence that the two-state model does not account for savings

b. Sing and Smith, 2010 when describing how the two-state model may account for interference

c. With reference to (b) the authors might also consider Lerner et al., 2019 (bioRxiv) for a more recent discussion of interference

5. In terms of other citations, the authors might consider citing Heald et al., 2018 when describing dual adaptation and contextual cues.
