# Supplementary material for: Timescales of motor memory formation in dual-adaptation
Source: PLoS Comput Biol. 2020 Oct 19;16(10):e1008373. doi: 10.1371/journal.pcbi.1008373 (PMC7595703; doi:10.1371/journal.pcbi.1008373)
Supplement: Attachment — Submitted filename: comments.docx [file pcbi.1008373.s012.docx]

I appreciate that the authors so thoroughly addressed my comments in the revised manuscript. I am satisfied with their analysis and have no further major comments. Here is one minor issue.

Perhaps I am missing something, but I am confused about Fig. 5G. On line 331, the authors state “when fitting both models (weighted switch) to the two groups of participants…”. I am confused about what is different about the fitting procedure you are alluding to on line 331, versus what you did to select these groups in the first place? Are you fitting the mean behavior across the 3 participants and 7 participants? I’m trying to figure this out but can’t seem to find this info in the Methods of the Figure legend. Overall, I think Lines 331-336 could be a bit better explained because I am confused why participants who were “dual-rate best-fit” are now “triple-rate best-fit” when examining the bar plot in Fig. 5G.
